# Synthesis and Characterization of Al- and SnO_2_-Doped ZnO Thermoelectric Thin Films

**DOI:** 10.3390/ma14226929

**Published:** 2021-11-16

**Authors:** Giovanna Latronico, Saurabh Singh, Paolo Mele, Abdalla Darwish, Sergey Sarkisov, Sian Wei Pan, Yukihiro Kawamura, Chihiro Sekine, Takahiro Baba, Takao Mori, Tsunehiro Takeuchi, Ataru Ichinose, Simeon Wilson

**Affiliations:** 1 College of Engineering, Shibaura Institute of Technology, 307 Fukasaku, Minuma-ku, Saitama 337-8570, Saitama Prefecture, Japan; 2 Toyota Technological Institute, Tempaku Ward, Hisakata 2-12-1, Nagoya 468-8511, Aichi Prefecture, Japan; saurabhsingh@toyota-ti.ac.jp (S.S.); t_takeuchi@toyota-ti.ac.jp (T.T.); 3 Physics and Engineering Department, Dillard University, 2601 Gentilly Blvd, New Orleans, LA 70122, USA; adarwish@dillard.edu (A.D.); Swilson@dillard.edu (S.W.); 4 SSS Optical Technologies, 515 Sparkman Drive, Huntsville, AL 35816, USA; Mazillo123@yahoo.com; 5 Muroran Institute of Technology, 27-1 Mizumotocho, Muroran 050-8585, Hokkaido Prefecture, Japan; 21043057@mmm.muroran-it.ac.jp (S.W.P.); y_kawamura@mmm.muroran-it.ac.jp (Y.K.); sekine@mmm.muroran-it.ac.jp (C.S.); 6 International Center for Materials Nanoarchitectonics (WPI-MANA), National Institute for Materials Science (NIMS), Namiki 1-1, Tsukuba 305-0044, Ibaraki Prefecture, Japan; BABA.Takahiro@nims.go.jp (T.B.); MORI.Takao@nims.go.jp (T.M.); 7 Graduate School of Pure and Applied Sciences, University of Tsukuba, 1-1-1 Tennodai, Tsukuba 305-8577, Ibaraki Prefecture, Japan; 8 Central Research Institute of Electric Power Industry (CRIEPI), 2-6-1 Nagasaka, Yokosuka 240-0196, Kanagawa Prefecture, Japan; ai@criepi.denken.or.jp

**Keywords:** thermoelectricity, Seebeck coefficient, thermal conductivity, thin film, oxides

## Abstract

The effect of SnO_2_ addition (0, 1, 2, 4 wt.%) on thermoelectric properties of *c*-axis oriented Al-doped ZnO thin films (AZO) fabricated by pulsed laser deposition on silica and Al_2_O_3_ substrates was investigated. The best thermoelectric performance was obtained on the AZO + 2% SnO_2_ thin film grown on silica, with a power factor (*PF*) of 211.8 μW/m·K^2^ at 573 K and a room-temperature (300 K) thermal conductivity of 8.56 W/m·K. *PF* was of the same order of magnitude as the value reported for typical AZO bulk material at the same measurement conditions (340 μW/m·K^2^) while thermal conductivity *κ* was reduced about four times.

## 1. Introduction

The increasing worldwide demand for energy and the resultant depletion of fossil fuels have brought new challenges for the scientific community [1]. One of the major issues is to develop high-efficiency devices for capturing energy from abundant natural sources such as solar, wind and geothermal energy. Another surplus, but mostly unused, source of energy is wasted heat. There are huge waste heat sources in our environments covering a wide range of temperatures (300~1200 K): industrial processes, domestic stoves and radiators, electrical lighting, pipelines, electrical substations, subway networks, automotive exhaust tubes, but also geothermal heat, body heat, and so on: about 66% of the annual world energy consumption is lost as waste heat, and the loss corresponds to the stellar amount of 3·10^20^ J per year, just considering the past 10 years [2,3]. A highly promising method for energy recovery from such heat sources is the utilization of thermoelectric (TE) materials that can convert various types of waste heat flow into electricity. The possibility of conversion of heat in electricity was discovered in 1821 by Thomas Seebeck [4]: a junction of two metals generates the thermoelectric voltage Δ*V* when a temperature gradient Δ*T* is created across it. In mathematical form it can be expressed as:

Δ*V* = *S* · Δ*T* (1)

where *S* is the material-dependent Seebeck coefficient. The performance of any TE material is quantified by the figure of merit (*ZT*):

(2) $$ZT=\frac{\sigma S^{2}}{\left(\kappa_{el}+\kappa_{ph}\right)}T$$

where *σ* is the electrical conductivity, *S* the Seebeck coefficient, *T* the absolute temperature, *κ_el_* the electrical thermal conductivity and *κ_ph_* the phononic thermal conductivity being *κ* = *κ_el_ + κ_ph_* the total thermal conductivity.

*ZT* is related to the efficiency *η* of heat/electrical energy conversion by the relation:

(3) $$\eta =\frac{∆T}{T_{H}}\left(\frac{\sqrt{1+ZT_{ave}}-1}{\sqrt{1+ZT_{ave}}+\frac{T_{C}}{T_{H}}}\right)$$

State-of-the-art TE materials for heat conversion can operate at *T* = 300~1200 K, with *ZT* = 0.1~2.6, corresponding to *η* = 1~20%. Therefore, after two centuries, TE devices are seldom utilized in daily life. TE modules have obtained brilliant success, however, in niche applications, for example for powering the space probe Cassini [5], while high-conversion modules to harvest waste heat of car engines remained at the state of prototypes [6]. *ZT* and *η* must be strongly improved to make TE power competitive with the common thermodynamic cycles based on fossil fuel burning, solar conversion and nuclear power plants. The improvement of *ZT* can be obtained by enhancing *σ* and/or *S*, or the product *σS*^2^ which is called “power factor” (*PF*), and/or by decreasing the total thermal conductivity. However, *κ_el_* = *LTσ*, with *L* = 2.44 *×* 10^−8^ W·Ω·K^−2^ is the Lorentz number while *κ_ph_* = 1/3 *CvЛ* where *C* is the heat capacity, *v* is the speed of phonons, *Л* is the phonon mean free path. It is thus necessary to decouple *σ* and *κ_el_* and work on the depression of *κ_ph_*.

Since the discovery of the Seebeck effect, the benefit of TE harvesting has been understood and a wealth of TE materials has been discovered. After the discovery of BiTe with *ZT* = 0.5 [7] in 1954, the *ZT* of TE materials did not improve for a very long time. In 1993, a seminal paper [8] theoretically predicted the drastic depression of *κ_ph_* due to reduction of the mean free path of phonons (*Л*) to a few nanometers, which resulted in substantial improvement of *ZT*. This concept was firstly validated in Bi_2_Te_3_/Sb_2_Te_3_ multilayer thin films incorporating natural nano-sized precipitates, showing the extremely small *κ* value (0.22 W·m^−1^ K^−1^) and a huge value of *ZT* = 2.5 at T = 300 K [9].

These results were due to the scattering of phonons by nano-defects spontaneously formed inside the thin films. However, TE harvesters based on BiTe and related materials cannot be produced on a large scale because they contain rare (Te) and poisonous (Sb, Bi, Pb) elements, and require high-cost processing. Though the last ten years a great progress has been made with *ZT* = 2.6 for SnSe single crystals [10] and 3.1 for polycrystalline SnSe [11], there is still room for finding even more efficient TE materials in a wide T region.

In particular, the attention of researchers has been focused on the development of stable, environmentally benign, abundant, and cost-effective TE materials based on oxides. In the 1990s, extensive research in the area of bulk oxides focused on the enhancement of the TE performance using atomic substitutions and improved grain connection [12,13,14,15]. To date, the best TE performance of the currently available oxide materials is *ZT* = 0.64 for *n*-type Zn_0.96_Al_0.02_Ga_0.02_O [13] and 0.74 for *p*-type Ca_2.5_Tb_0.5_Co_4_O_9_ [14] at 1000 K. The *ZT* of oxides is not yet up to the level of the best conventional TE materials and needs to be drastically improved to be acceptable for practical applications. The bulk oxides also have the main disadvantages of requiring a long time for sintering and fabrication of the *n*- and *p*- elements and their assembly in modular shape, and mechanical fragility. All these drawbacks can be overcome by using oxide thin films. Thin-films have significant advantages, such as low dimensionality, rapid fabrication, control of strain at the interface with substrates, and the possibility to insert artificial nano-defects to improve the phonon scattering. Thermoelectric thin films are also attractive for their applicative potential for energy harvesting to power Internet of Things (IoT) sensors [15,16].

Recently, our group studied oxide-thin TE films and obtained encouraging preliminary results. First, epitaxial thin films of 2% Al-doped ZnO (AZO) were fabricated by pulsed laser deposition (PLD) on several single crystal (SrTiO_3_, Al_2_O_3_) and amorphous (silica) substrates. Regardless of a particular substrate, the films always showed higher values of *ZT* in comparison with the corresponding bulk AZO: for example, at *T* = 600 K, *ZT_AZO-on-STO_* = 0.03 while *ZT_bulk_* = 0.014 [17,18]. The superior performance of films is due to their lower thermal conductivity: *κ_AZO-on-STO_* (300 K) = 6.5 W/m·K [16,17] while *κ_bulk_* (300 K) = 34 W/m·K. In these series, the grain boundaries can be considered as natural nanodefects for an enhanced scattering of phonons and consequent decrease of *κ*. As a demonstration of this effect, the film on fused silica, showing additional grain boundaries at the seed layer on the substrate, had even lower thermal conductivity: *κ_silica_* (300 K) = 4.89 W/m·K and larger *ZT*: *ZT_silica_* (600 K) = 0.045 [18].

The insertion of artificial nanodefects has been subsequently considered with the purpose of further reduction of *κ* and enhancing *ZT*. Several approaches have been tried by our group: insertion of hydroquinone nanolayers in AZO films prepared by atomic layer deposition (ALD): *κ_ALD_* (300 K) = 3.56 W/m·K [19]; addition of polymethylmethacrylate (PMMA) particles to AZO films prepared by multi-beam multi-target matrix-assisted PLD (MBMT/MAPLE-PLD): *κ_MAPLE_* (300 K) = 5.9 W/m·K and *ZT_MAPLE_* (600 K) = 0.0061 [20]; formation of nanopores in AZO films prepared by mist-chemical vapor deposition (mist-CVD): *κ_porous_* (300 K) = 0.60 W/m·K and *ZT_porous_* (300 K) = 0.057 [21]; dispersion of Al_2_O_3_ nanoparticulate in AZO films prepared by surface-modified target PLD: *κ_nanoAl2O3_* (300 K) = 3.98 W/m·K and *ZT_nanoAl2O3_* (600 K) = 0.0007 [22]. Several groups worldwide have recently obtained excellent results with different kinds of dopant, like the *κ* (300 K) = 1.19 W/m·K and *ZT* (300 K) = 0.1 of magnetron sputtered AZO films by Loureiro et al. [23], *κ* (323 K) = 3.37 W/m·K and *ZT* (423 K) = 0.052 of CNT-added evaporated porous AZO films by Liu et al. [24], the *κ* (300 K) = 1.1 W/m·K and *ZT* (300 K) = 0.042 of amorphous ZnO*_x_*N*_y_* PLD films by Hirose et al. [25], the *κ* (300 K) = 1.8 W/m·K and *ZT* (383 K) = 0.019 of dual-doped AlGaZnO PLD films by Nguyen, et al. [26], just to cite the most impressive. All these successful examples highlight the promise of nanostructured doped ZnO films for future energy-harvesting applications.

In this work, we have focused on enhancing the thermoelectric performance of Al-doped ZnO films by the addition of a controlled amount of SnO_2_ as a secondary dopant. AZO has the same structure of hexagonal ZnO: wurtzite, hexagonal, space group ***P6_3_mc***, cell parameters *a* = *b* = 0.3289 nm and *c* = 0.5307 nm, while SnO_2_ is cassiterite, tetragonal, space group ***P4_2_/mnm*** with cell parameters *a* = *b* = 0.4832 nm and *c* = 0.3243 nm. Since the mismatch between the *a*-axis of AZO and the *c*-axis of SnO_2_ is 1.4%, we forecasted the growth of quasi-epitaxial nanostructures (nanoparticles) of SnO_2_ with *c*_SnO2_//*a*_AZO_, dispersed in the AZO matrix.

The motivation of this work is to prove the presence of SnO_2_ nanoparticulates in the AZO matrix and verify the optimal content of SnO_2_ for the TE properties of the nanocomposite Al- and SnO_2_-doped (AZO + SnO_2_) thin films.

## 2. Materials and Methods

Two sets of four thin films were prepared using various compositions and substrate materials. All samples were deposited both on amorphous silica and *c*-axis oriented alumina by pulsed laser deposition (PLD) technique focusing a Nd:YAG (266 nm, 10 Hz) laser on dense pellets. Four commercial targets (Kurt Lesker Ltd., Jefferson Hills, PA, USA) used for film making had different contents of SnO_2_ (0, 1, 2 and 4 wt.%) dispersed in the Al-ZnO (AZO) main phase where the Al content was kept as 2 wt.%. Prior to the deposition, the substrates were cleaned at 773 K for 2 h, then glued by conductive silver glue to a rectangular Inconel plate, which was put in direct thermal contact with a cartridge heater and finally inserted into the vacuum chamber. All films were grown with an energy density of about 4.2 J/cm^2^ for 60 min at 573 K, in the atmosphere of 20 mtorr of oxygen. The samples deposited on silica are named AZO_xS and the ones deposited on alumina AZO_xA, with x = 0, 1, 2 and 4 referring to the concentration (wt.%) of the SnO_2_ in the respective targets.

Electrical conductivity (*σ*) and Seebeck coefficient (*S*) were measured by means of the four-probe method using a ZEM-3 (ULVAC Advance-Riko, Yokohama, Kanagawa, Japan) apparatus under partial He pressure to assure thermal transport between the heater and the sample. Measurements with ZEM-3 were conducted performing three cycles between 353 and 573 K and measuring both heating and cooling processes. The *σ* and *S* curves reported in Section 3.2 for each sample were those obtained from the last cooling cycle, considered as the most stable and reliable. The uncertainties of the ZEM-3 measurements were ±4% for the electrical conductivity and ±3% for the Seebeck coefficient.

For comparison and validation of data, some selected samples were also characterized by a custom-made apparatus whose working principle and measurement protocol are reported elsewhere [27,28]. The error in the thermoelectric coefficients measurements is ±5% in the case of σ, and ±7% for *S*.

The thickness of the thin films (in the range 295–585 nm, as summarized in Table 1) was measured using a Dektak 6M Stylus Profiler (Bruker, Billerica, MA, USA) and a Filmetrics Profilm 3D (KLA, Milpitas, CA, USA).

The X-ray diffraction analysis was conducted using a Bragg-Brentano powder diffractometer (Smart Lab 3, Rigaku Corporation, Tokyo, Japan) using the Cu K_α_ radiation in the 10–100° angular range with an angular step of 0.02° (power settings: 40 mA, 40 kV).

The TEM images and EDS analysis were conducted using a JEM-2100F (JEOL, Akishima, Tokyo, Japan) with EDS spectroscopy. The STEM beam size was set to 0.5 nm.

The picosecond time-domain thermo-reflectance (TD-TR) technique using a customized system PicoTR (PicoTherm, Netzsch Japan KK, Tsukuba, Japan) was utilized to measure the thermal conductivity of the samples at room temperature (T = 300 K) in the cross-plane direction.

Measurement of Hall coefficient at room temperature was performed using a standard four-terminal method with a commercial PPMS instrument (Quantum Design, San Diego, CA, USA) at an AC current of 5 mA.

## 3. Results and Discussion

### 3.1. Structural and Morphological Characterization

**Figure 1:**
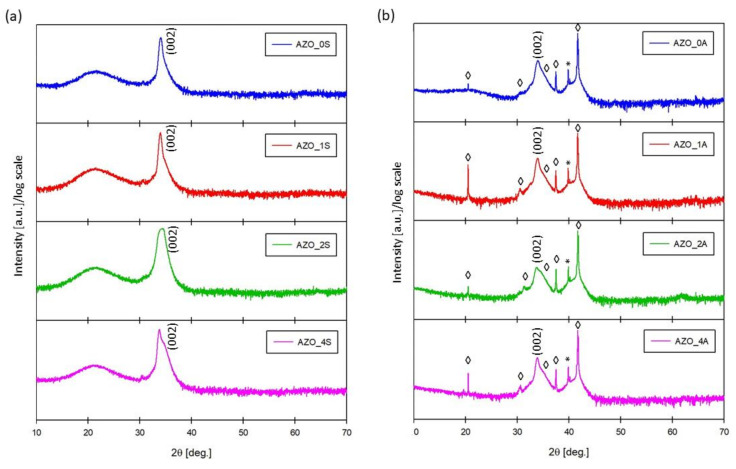
X-ray diffraction (XRD) spectra of Al-doped ZnO (AZO) films deposited (**a**) on silica (series AZO_xS); (**b**) on Al_2_O_3_ (series AZO_xA). *(hkl)* reflections of AZO [18] are indicated, while ◊ labels the peaks of Al_2_O_3_ substrate and asterisks * the unattributed peaks.

The XRD spectra of the thin films fabricated on amorphous silica and single-crystal c-axis oriented Al_2_O_3_ are presented in Figure 1a,b, respectively.

On both substrates, the preferred orientation of AZO films was along the c-axis since only (00l) reflection appeared. The presence of SnO_2_ in the films of both series was undetectable by X-rays even for the highest amount (x = 4).

**Figure 2:**
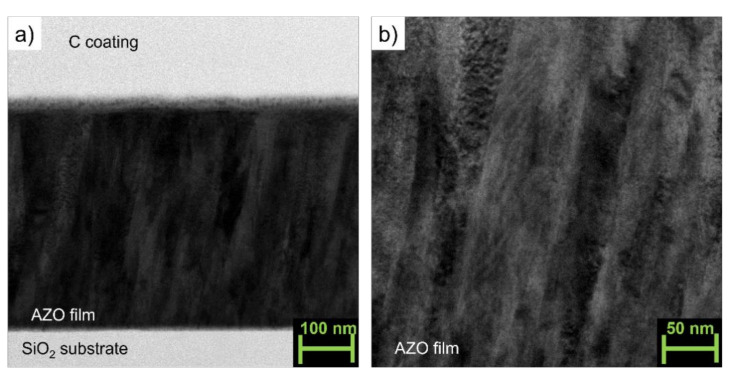
(**a**) Cross-sectional transmission electron microscope (TEM) image (magnification 150,000) of the AZO/silica thin film AZO_0S; (**b**) double magnification (300,000×) taken in the central zone of Figure 2a.

**Figure 3:**
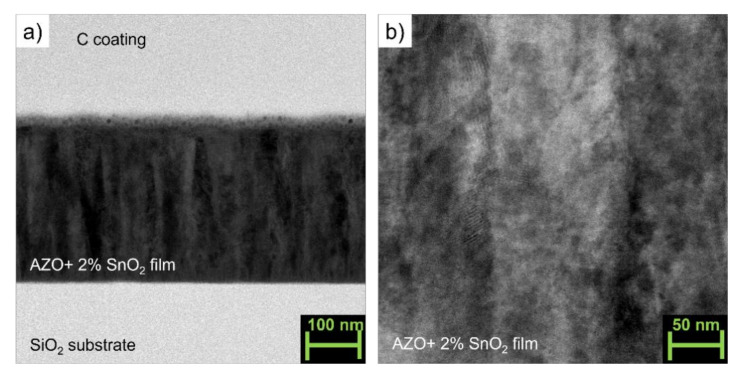
(**a**) Cross-sectional TEM image (magnification 150,000) of the AZO/silica thin film AZO_2S; (**b**) double magnification (300,000×) taken in the central zone of Figure 3a.

The cross-sectional TEM images (magnification 150,000, Figure 2a and Figure 3a of two representative samples deposited on fused silica without (AZO_0S) and with the addition of SnO_2_ (2 wt.%, AZO_2S) reveal a typical columnar growth along the c-axis of the films as often reported in the literature for AZO PLD thin films [16,17,19,21]. The addition of SnO_2_ (AZO_2S) did not affect the morphology of the films. The higher magnification images (300,000×, Figure 2b and Figure 3b for AZO_0S and AZO_2S, respectively) show that the columns were well connected without the presence of pores or misoriented grains so that the density of the films could be considered close to the theoretical value.

**Figure 4:**
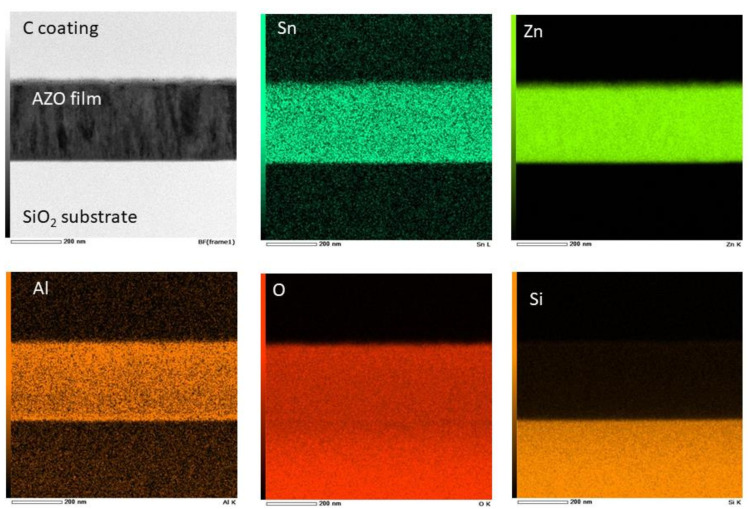
TEM-EDS (energy-dispersive spectroscopy) elemental maps of the AZO + SnO_2_ 2 wt.% silica thin film (AZO_2S) for the atoms Sn, Zn, Al, O and Si.

The TEM-EDS mapping performed on the sample AZO_2S (Figure 4) reveals a uniform distribution not only of the elements Al, Zn, O, but also of Sn. Contrary to the predictions based on the 1.4% lattice mismatch, Sn did not form nanoscale aggregates in the AZO matrix, but presumably entered as substitutional or interstitial atoms in the AZO elementary cell. According with TEM-EDS analysis, the atomic percentage of Sn in the film prepared with 2 wt.% SnO_2_-added AZO target is 0.49%. The atom % of oxygen is 33.91%. Since it is not possible to separate the contribution of oxygen from SnO_2_ and from ZnO, we can only compare the percentage of Sn in the target and in the film. The percentage of Sn in SnO_2_ is 78.77%, which in 2 wt.% SnO_2_ corresponds to 1.58%. This means that in the film the amount of Sn is about 1/3 than in the target. Si is not considered in the discussion being part of the substrate only.

### 3.2. Transport and Thermoelectric Characterization

**Figure 5:**
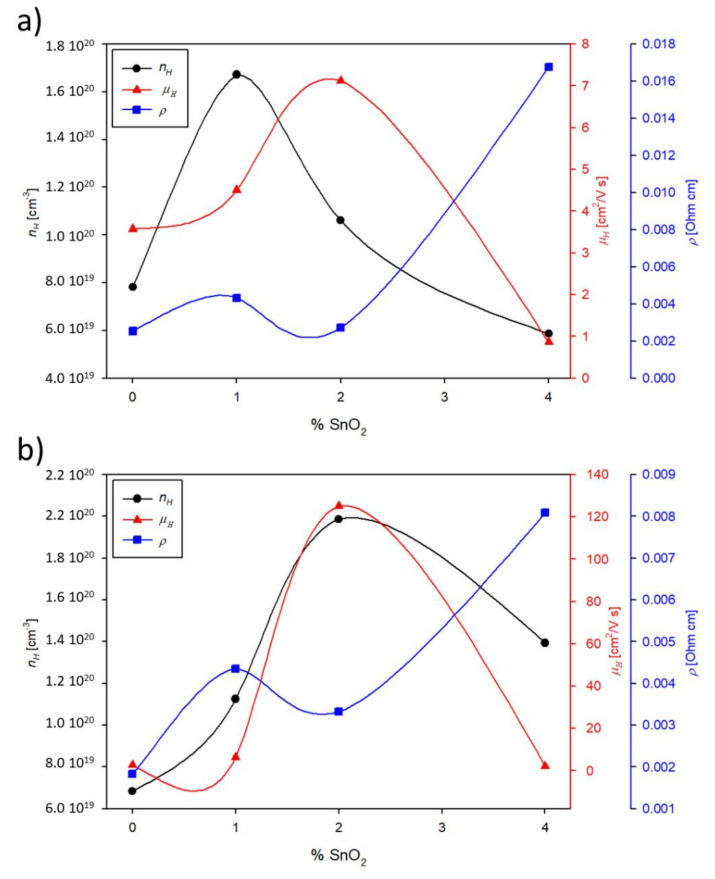
Hall effect measurements of the number of carriers and their mobility for films deposited (**a**) on silica (series AZO_xS) and (**b**) Al_2_O_3_ (series AZO_xA) plotted versus the % content of SnO_2_ (x). Note that reported is the absolute value of *n_H_* with the aim of simplifying the reading. The resistivity of both series was measured at the lowest temperature (348 K) using the ZEM-3 apparatus.

Room temperature Hall measurement was taken to find the carriers’ concentration *n_H_* and mobility *μ_H_* values for the AZO_xS and AZO_xA films. The results are in Figure 5, together with room temperature resistivity values from the ZEM-3 analysis.

The resistivity at room temperature has a clear ascending trend with the increase of the content of SnO_2_ for both substrates. The films on Al_2_O_3_ had half the resistivity of those on silica. The range of *n_H_* was almost the same for two substrates, and of the order of 10^20^ cm^−3^, as expected for a typical semiconductor such as AZO. The maximum number of carriers occurred at x = 1 on silica and at x = 2 on Al_2_O_3_. The carrier mobility had the maximum at x = 2 on both substrates but was about 20 times larger for AZO_2A than for AZO_2S. AZO_0S had greater mobility than AZO_0A and this could be explained by the larger misfit dislocations generated on Al_2_O_3_, due to a lattice misfit of 15% [18,29]. Surprisingly, by increasing the content of SnO_2_ doping, there was a reversal trend and the films grown on Al_2_O_3_ presented higher mobility, with the maximum value for AZO_2A-20 times larger than for AZO_0S.

**Figure 6:**
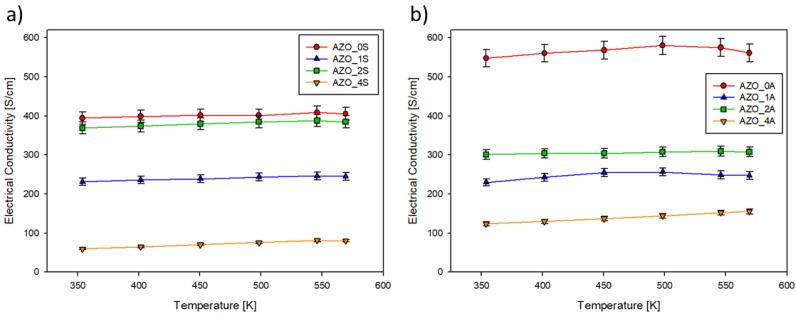
Electrical conductivity plotted vs. temperature of thin films deposited on (**a**) silica (series AZO_xS); (**b**) on Al_2_O_3_ (series AZO_xA).

The electrical conductivity (*σ*) for the AZO films is presented in Figure 6 as a function of temperature in the range 300–600 K. Figure 6 shows that *σ* increases with rising temperature, confirming the semiconductor behaviour of the samples. On both substrates, the film grown without additional SnO_2_ had the largest *σ* followed by the film with x = 2. 

**Figure 7:**
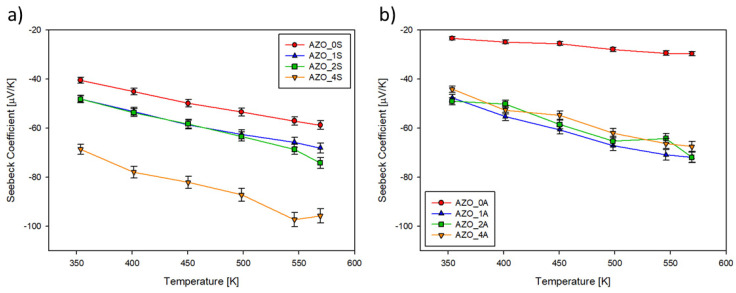
Seebeck coefficient (*S*) plotted vs. temperature of thin films deposited on (**a**) silica (series AZO_xS); (**b**) on Al_2_O_3_ (series AZO_xA).

The temperature dependence (300–600 K) of Seebeck coefficient (*S*) of the AZO films is plotted in Figure 7. S had a negative sign, confirming the *n*-type conductivity of AZO. Figure 7 shows that *S* decreases with the rising temperature, a reversal trend with respect to *σ* as commonly expected for semiconductor materials. On both substrates, the pure AZO film had the lowest *S* while the sample fabricated with a target containing 4% of SnO_2_ presents the largest one.

The Seebeck coefficient can be expressed as [30]:

(4) $$S=\left(\frac{8\pi^{2}k_{B}^{2}}{3eh^{2}}\right)m^{*}T{\left(\frac{}{3n}\right)}^{2/3}$$

where *k_B_* is Boltzmann’s constant, *h* is Planck’s constant, *T* is the absolute temperature, m* is the effective mass and *n* is the carrier concentration. The effective mass is also expressed as m* = *α m_e,_* where *m_e_* is the mass of the electron and *α* is a positive rational number.

**Figure 8:**
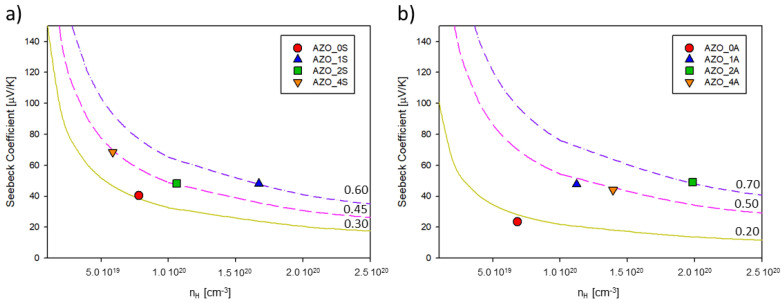
Pisarenko plot at 300 K for thin films deposited on (**a**) silica (series AZO_xS) and (**b**) on Al_2_O_3_ (series AZO_xA).

The room temperature (300 K) “Pisarenko plots” (*S* versus *n_H_*) for both series of films are presented in Figure 8. According to the graphs, m* was (0.3–0.6) *m_e_* and (0.2–0.7) *m_e_* for the films grown on silica and alumina, respectively. The fractional me was consistent with the parabolic band approximation and relatively simple band structure reported for ZnO [31].

**Figure 9:**
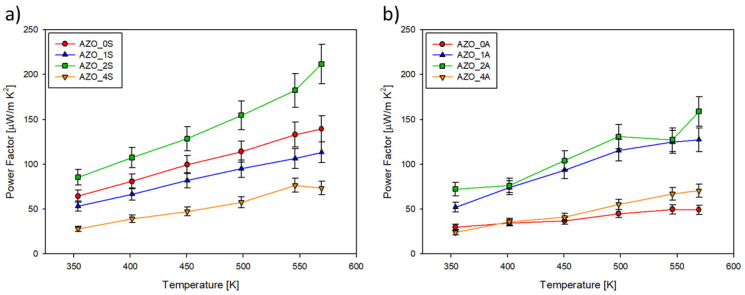
Power factor *PF* = *σS*^2^ plotted vs. temperature for thin films deposited on (**a**) silica (series AZO_xS) and (**b**) on Al_2_O_3_ (series AZO_xA).

The calculated power factor (*PF*) as a function of temperature is shown in Figure 9. The maximum power factor value was found for sample AZO_2S at 573K as 211.78 mW/m·K^2^. This value was of the same order of magnitude as that measured for bulk AZO at the same conditions (−340 mW/m·K^2^) [32] and was also comparable with the values reported for AZO thin films [17,18,23,24,25,26].

**Figure 10:**
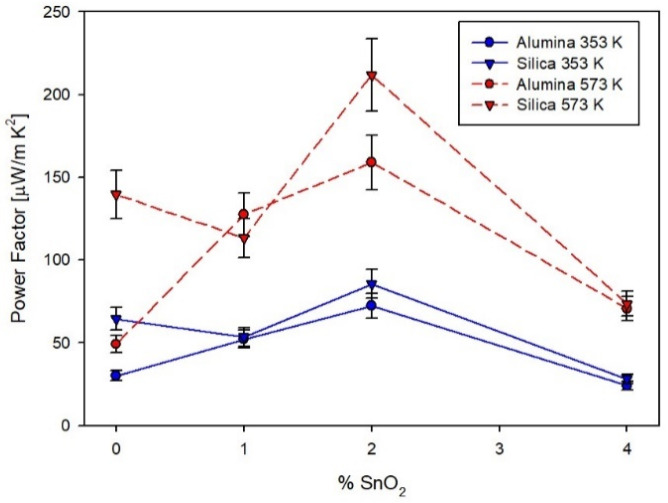
The trend of power factor with the content of SnO_2_ for the two series of films on silica and alumina.

The *PF*s of all films at 353 K and 573 K are summarized in Figure 10, making clear that on both substrates, 2% of SnO_2_ represents the optimal dopant concentration.

For the films AZO_0S and AZO_2S deposited on silica the measurements were repeated using two different apparatuses to verify the accuracy of the reproducibility of data. The comparison between the pair sets of data for *σ*, *S* and *PF* is reported in Appendix C.

The transport and thermoelectric properties of the films are summarized in Table 1.

The room temperature (300 K) thermal conductivity (*κ*) of the sample with the highest *PF* (AZO_2S) and of a reference sample from the same batch (AZO_0S) was evaluated by the TD-TR method [32,33,34,35] according to the details described in Appendix A. The value of *κ* was 8.88 and 8.56 W/m·K for samples AZO_0S and AZO_2S, respectively. These values were in the same range as those reported for pure AZO films deposited by PLD [16,17,21]. Therefore, the four-fold decrease of *κ* with respect to bulk [32] must be attributed to the phonon scattering of grain boundaries, and not to the addition of SnO_2_. The calculation of *ZT* (*T* = 353 K) using Equation (2) gave the following result: *ZT* (AZO_0S) = 0.003 and *ZT* (AZO_2S) = 0.004, of the same order as for AZO thin film deposited by PLD on different substrates. Conservative estimates of *ZT′* (*T′* = 573 K) were *ZT′* (AZO_0S) = 0.009 and *ZT′* (AZO_2S) = 0.014. Since for oxide thin films *κ* is expected to decrease with *T* [33], *ZT* was calculated using a conservative approach from the *PF* values at 353 K or 573 K, *κ* at 300 K, and *T* = 353 K or 573 K.

These values of *ZT* did not surpass the reported performance of bulk AZO at the same conditions and were also of the same or lower level as for other AZO films prepared by PLD. This indicated that SnO_2_ could not be considered as an optimal dopant for AZO thin films for thermoelectric applications. On the other hand, it could be that the fine dispersion of SnO_2_ into AZO matrix enhanced the photoelectric response of AZO, as reported for SnO_2_/ZnO hierarchical nanostructures prepared by the electrospinning method [34], or could be used to fabricate compact gas sensors, as reported for ZnO–SnO_2_ nanofibers [35].

## 4. Summary

In summary, we fabricated using pulsed laser deposition (PLD) two series of thermoelectric thin films of Al-doped ZnO (AZO) doped with various concentrations of SnO_2_ (x = 0, 1, 2, 4 wt.%) on the substrates made of fused silica and Al_2_O_3_ (001) single crystals. The goal was to enhance the thermoelectric performance. All the films were c-axis oriented. The films deposited on silica showed the highest values of Seebeck coefficient (*S*) and power factor *(PF)* in comparison with the films on Al_2_O_3_ with the same content of SnO_2_. The AZO film on silica with x = 2 showed the best performance, with *σ* = 385.0 S/cm, *S* = –74.17 mV/K, and *PF* = 211.8 μW/m·K^2^ at the maximum operating temperature (573 K). The room temperature (300 K) thermal conductivity of this sample was evaluated as *κ* = 8.56 W/m·K, with a calculated figure of merit *ZT* (353 K) = 0.003 and a projected value of *ZT* (573 K) = 0.014, comparable with *ZT* of the nanostructured PLD AZO films grown on several substrates.

## Figures and Tables

**Table 1:** Thickness, electrical and thermoelectric properties of the AZO films deposited on silica (series AZO_xS) and on Al_2_O_3_ (series AZO_xA) for different concentrations x (%) of SnO_2_ dopant.

Sample	Thickness [nm]	*n*_H_ [cm^−3^]	*μ*_H_ [cm^2^/V·s]	*σ* [S/cm]		S [μV/K]		PF [μW/m·K^2^]		k [W/m·K]
		(300 K)	(300 K)	(353 K)	(573K)	(353 K)	(573K)	(353 K)	(573K)	(300 K)
AZO_0S	595	−7.812·10^19^	3.571	394.7	405.3	−40.41	−58.66	64.47	139.5	8.88
AZO_1S	505	−1.671·10^20^	4.503	231.2	245.0	−48.07	−68.02	53.43	113.4	-
AZO_2S	300	−1.061·10^20^	7.119	369.2	385.0	−48.13	−74.17	85.54	211.8	8.56
AZO_4S	295	−5.865·10^19^	0.8606	59.63	80.23	−68.57	−95.73	28.03	70.70	-
AZO_0A	535	−6.832·10^19^	2.576	547.8	561.4	−23.38	−29.61	29.94	49.21	-
AZO_1A	498	−1.124·10^20^	6.345	229.8	247.7	−47.66	−71.72	52.19	127.4	-
AZO_2A	535	−1.986·10^20^	125.1	301.4	307.6	−49.00	−71.87	72.38	158.9	-
AZO_4A	480	−1.394·10^20^	2.143	123.7	155.4	−44.06	−67.45	24.01	70.70	-

**Figure A1:**
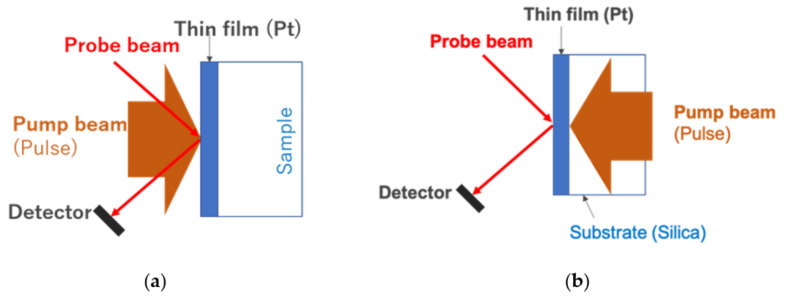
Diagram of thermo-reflectance measurement with (**a**) front-detect (FF) configuration and (**b**) rear-heat front-detect (RF) configuration.

**Figure A2:**
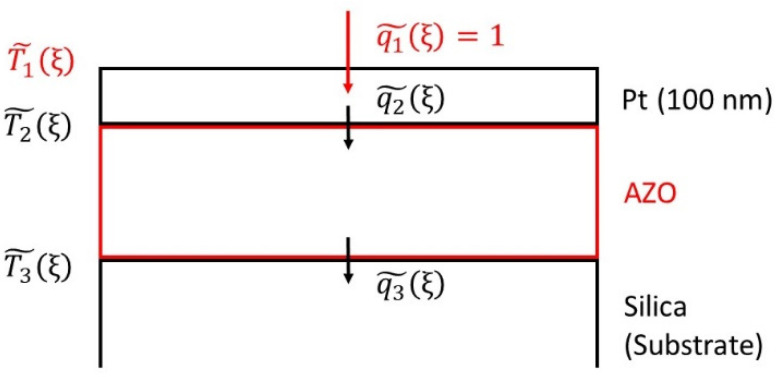
Diagram of the applied model.

**Figure A3:**
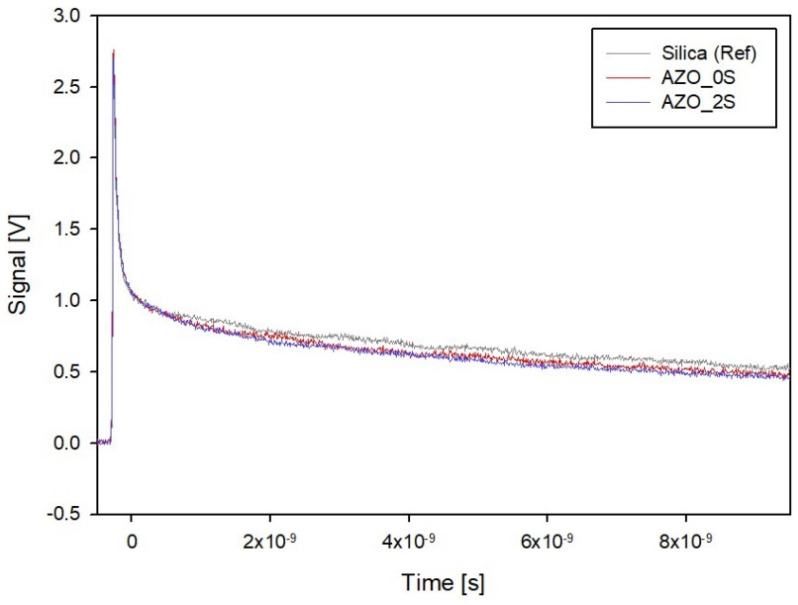
Thermo-reflectance signals of the silica reference and AZO samples.

**Figure B1:**
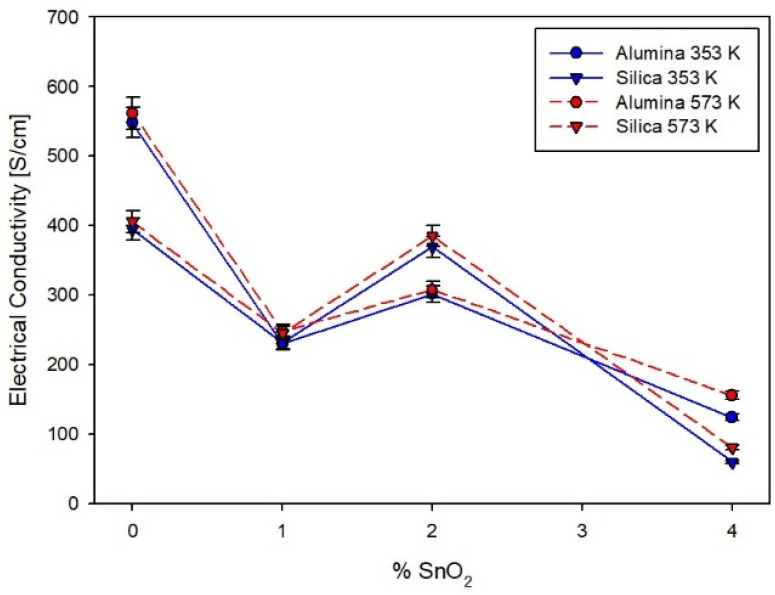
Electrical conductivity plotted versus the concentration of SnO_2_ for the two series of films on silica and alumina.

**Figure B2:**
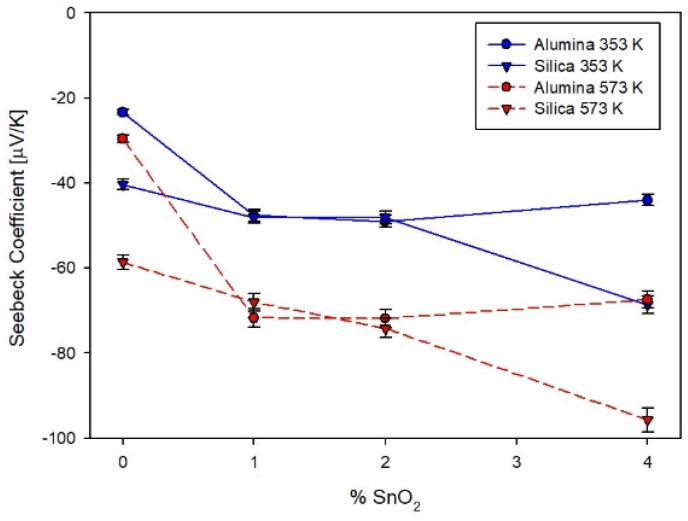
Seebeck coefficient plotted versus the concentration of SnO_2_ for the two series of films on silica and alumina.

**Figure C1:**
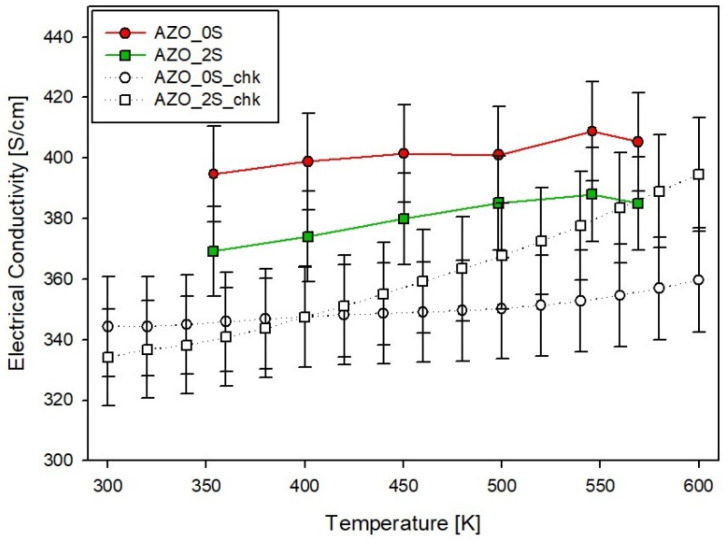
Electrical conductivity plotted vs. temperature of AZO_0S and AZO_2S obtained with two different setups. Filled symbols are the same as those reported in the plots in the main text, while the open symbols “chk” were obtained with another device.

**Figure C2:**
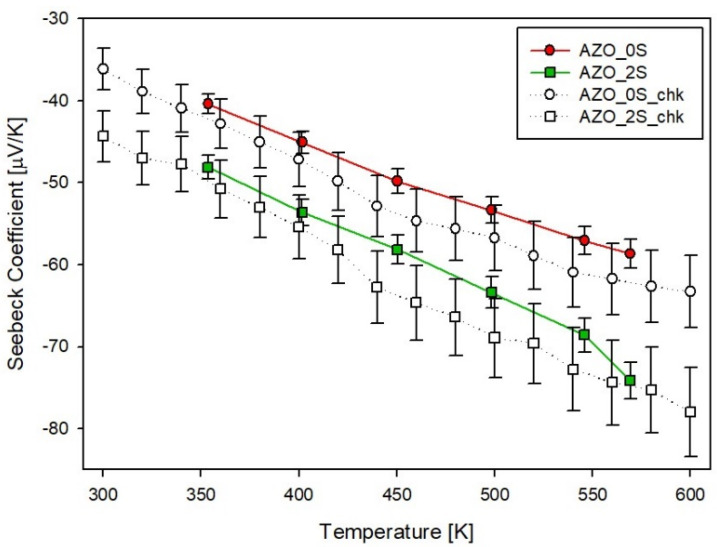
Seebeck coefficient plotted vs. temperature of AZO_0S and AZO_2S obtained with two different setups. Filled symbols are the same as those reported in the plots in the main text, while the open symbols “chk” were obtained with another device.

**Figure C3:**
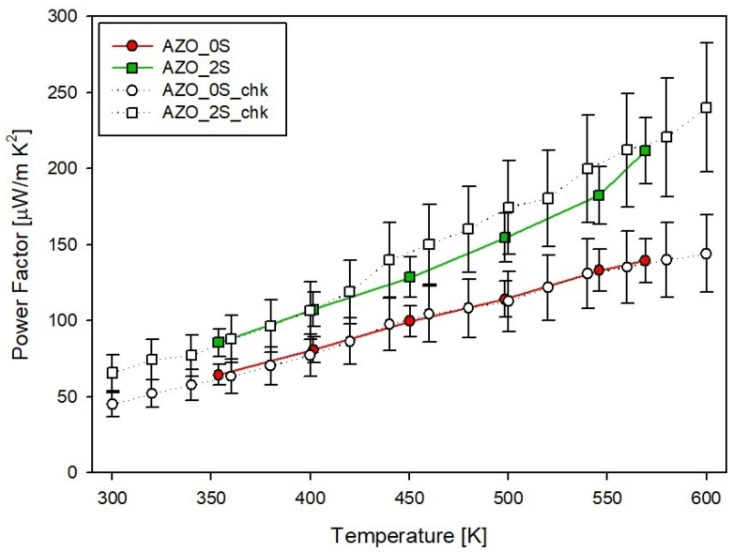
Power factor plotted vs. temperature of AZO_0S and AZO_2S calculated from experimental data obtained with two different setups. Filled symbols are the same as those reported in the plots in the main text, while the open symbols “chk” were obtained with another device.

## Data Availability

The data presented in this research study are available in this article.

## Appendix A

The picosecond time-domain thermo-reflectance (TD-TR) was utilized to measure the thermal conductivity of two samples in the cross-plane direction using the front-heat front-detect (FF) configuration. Prior to the measurements, a 100 nm layer of Pt was sputtered on the surfaces of the thin films (see Figure A1). As a reference sample, we used 100 nm-thick Pt film deposited on a silica substrate. Rear-heat front-detect (RF) configuration was used for the reference sample (see Figure A1b). Figure A3 shows the thermo-reflectance signal obtained from the reference sample. We determined 1946 [J/(s^0.5^ m^2^ K)] as the thermal effusivity of the silica substrate by curve fitting. (see Figure A1b).

After depositing the Pt layer on the surface, samples consisted of three layers (see Figure A2).

The thermal conductivities of the thin films were determined by the picosecond time-domain thermo-reflectance (TD-TR) technique, using the original focused beam system [36,37,38].

For the analysis, the mirror image method was used [39]. In the mirror image method, the temperature history of FF configuration could be described as:

(A1) $$T_{f}\left(t\right)=\frac{1}{b_{f}\sqrt{\pi t}}\left(1+2\overset{\infty }{\underset{n=1}{\sum }}\gamma^{n}\exp\left({-n}^{2}\frac{\tau_{f}}{t}\right)\right)\;$$

(A2) $$\tau_{f\;}=\frac{d_{f}{}^{2}}{\alpha_{f}}={\left(\frac{C_{f}}{b_{f}}\right)}^{2}\;$$

(A3) $$\gamma =\frac{b_{f}{\;-\;b}_{s}}{b_{f}{\;+\;b}_{s}}\;$$

where $\tau_{f}$ is the heat diffusion time (in Pt layer), γ is the dimensionless parameter, $b_{f}$ is the thermal effusivity of the Pt layer, $b_{s}$ is the thermal effusivity of the sample, $d_{f}$ is the thickness of the Pt layer and $\alpha_{f}$ is the thermal diffusivity of the Pt layer.

We already knew the value of $C_{f}$, thus we could determine the thermal effusivity of the sample $b_{s}$. We could also determine the thermal conductivity of the samples $\kappa_{s}$ using the following equation:

(A4) $$\kappa_{s}=\frac{b_{s}{}^{2}}{c_{s}\rho_{s}}\;\;$$

where *c_S_* and *ρ_S_* are the specific heat capacity and density of the AZO layer, respectively.

The aforementioned model assumed that the sample consisted of two layers and the second layer (AZO) was semi-infinite. This model could be derived from the relationship between the temperature and heat density of the sample layers using quadrupole matrix expression [39]:

(A5) $$\left[\begin{array}{c} {\tilde{q}}_{2}\left(\xi \right)\\ {\tilde{T}}_{2}\left(\xi \right) \end{array}\right]=\left[\begin{array}{cc} \cosh\sqrt{\tau_{1}\xi } & -b_{f}\sqrt{\xi }\sinh\sqrt{\tau_{1}\xi }\\ -\frac{1}{b_{1}\sqrt{\xi }}\sinh\sqrt{\tau_{1}\xi } & \cosh\sqrt{\tau_{1}\xi } \end{array}\right]\left[\begin{array}{c} {\tilde{q}}_{1}\left(\xi \right)\\ {\tilde{T}}_{1}\left(\xi \right) \end{array}\right]\;{\tilde{T}}_{2}\left(\xi \right)=\frac{1}{b_{2}\sqrt{\xi }}{\tilde{q}}_{2}\left(\xi \right)$$

However, we found that the effect of the third layer (silica substrate) was significant, so we modified the model in order to account for the substrate. In the three-layered sample, quadrupole matrices were expressed as:

(A6) $$\left[\begin{array}{c} {\tilde{q}}_{3}\left(\xi \right)\\ {\tilde{T}}_{3}\left(\xi \right) \end{array}\right]=\left[\begin{array}{cc} \cosh\sqrt{\tau_{2}\xi } & -b_{2}\sqrt{\xi }\sinh\sqrt{\tau_{2}\xi }\\ -\frac{1}{b_{2}\sqrt{\xi }}\sinh\sqrt{\tau_{2}\xi } & \cosh\sqrt{\tau_{2}\xi } \end{array}\right]\left[\begin{array}{cc} \cosh\sqrt{\tau_{1}\xi } & -b_{1}\sqrt{\xi }\sinh\sqrt{\tau_{1}\xi }\\ -\frac{1}{b_{1}\sqrt{\xi }}\sinh\sqrt{\tau_{1}\xi } & \cosh\sqrt{\tau_{1}\xi } \end{array}\right]\left[\begin{array}{c} {\tilde{q}}_{1}\left(\xi \right)\\ {\tilde{T}}_{1}\left(\xi \right) \end{array}\right]\;{\tilde{T}}_{3}\left(\xi \right)=\frac{1}{b_{3}\sqrt{\xi }}{\tilde{q}}_{3}\left(\xi \right)\;\tau_{2}={\left(\frac{C_{2}}{b_{2}}\right)}^{2}$$

where *C_2_* is the heat capacity of the AZO layer, *b_3_* is the thermal effusivity of the silica substrate. We could derive the temperature response of the three-layered sample by solving this equation. This time we used the thermal diffusivity of the silica substrate determined using the reference sample (*b_3_* = 1946 J/(s^0.5^ m^2^ K).

Figure A3 shows the thermo-reflectance signal obtained from the samples. Table A1 shows the values determined by curve fitting.

**Table A1:** Thermal properties of samples AZO_0S and AZO_2S.

Sample	Heat Diffusion Time $\tau_{f}[s]$	γ	Thermal Effusivity $b_{s}{\;[J/(s}^{0.5}\cdot m^{2}\cdot K)]$	Thermal Conductivity $\kappa_{s}\;[W/(m\cdot K)]$
AZO_0S	5.19·10^−10^	0.541	3738	8.88
AZO_2S	5.24·10^−10^	0.529	3854	8.56

## Appendix B

The electrical conductivity *σ* is plotted versus the SnO_2_ concentration x in Figure B1.

The undoped sample had the highest *σ*, and x = 2 was the optimal dopant concentration.

The Seebeck coefficient *S* is plotted versus the concentration x of SnO_2_ in Figure B2.

The undoped sample had the lowest *S* while the sample with x = 4 had the largest *S*.

## Appendix C

The comparison of *σ*, *S* and *PF* curves obtained for the samples AZO_0S and AZO_2S is reported in the Figure C1, Figure C2 and Figure C3, respectively. Except for the case of s, the pair of curves are comparable within the experimental errors.
